# Keeping hospitals clean and safe without breaking the bank; summary of the Healthcare Cleaning Forum 2018

**DOI:** 10.1186/s13756-018-0420-3

**Published:** 2018-11-08

**Authors:** Alexandra Peters, Jon Otter, Andreea Moldovan, Pierre Parneix, Andreas Voss, Didier Pittet

**Affiliations:** 1 Infection Control Programme and WHO Collaborating Centre on Patient Safety, University of Geneva Hospitals and Faculty of Medicine, 4 Rue Gabrielle-Perret-Gentil, 1211 Geneva, Switzerland; 2 Imperial College London, London, UK; 3 Infection Prevention and Control Service, St. Constantin Hospital, Brasov, Romania; 4 Nouvelle Aquitaine Healthcare-Associated Infection Control Centre, Bordeaux University Hospital, Bordeaux, France; 5 Radboud Centre for Infections, Radboud UMC, and Department of Medical Microbiology, Canisius-Wilhelmina Hospital, Nijmegen, The Netherlands

**Keywords:** Infection prevention, Cleaning, Disinfection, Environment, Healthcare-associated infection, Public health, Environmental hygiene, Hand hygiene, Infection control, Antimicrobial resistance

## Abstract

Keeping hospitals clean is a crucial patient safety issue. The importance of the hospital environment in patient care has only recently been recognized widely in infection prevention and control (IPC). In order to create a movement for change, a group of international infection control experts teamed up with Interclean, the largest cleaning trade-show in the world to create the Healthcare Cleaning Forum. This paper is the result of this conference, which featured leaders in healthcare environmental science from across Europe.

Although the available literature is limited, there is now enough evidence to demonstrate that maintaining the hygiene of the hospital environment helps prevent infections. Still, good interventional studies are rare, the quality of products and methods available is heterogeneous, and environmental hygiene personnel is often relatively untrained, unmotivated, under-paid, and under-appreciated by other actors in the hospital. Coupled with understaffed environmental hygiene service departments, this creates lasting issues in regards to patient and healthcare worker safety.

The Healthcare Cleaning Forum was designed as a platform for healthcare experts, cleaning experts, hospital managers and industry to meet productively. The conference aimed to summarize the state-of-the-art knowledge in the field, create awareness and dialogue, challenge dogma and begin to shape a research agenda for developing the field of hospital hygiene and environmental control. Hospital environmental hygiene is far more complex than other types of cleaning; further evidence-based research in the field is needed. It involves the integration of current and new technologies with human elements that must work together synergistically to achieve optimal results. The education, training and career development, behavior, and work organization of environmental hygiene personnel are at the core of the proposals for the creation of a global initiative. Ultimately, what is needed is a reevaluation of how hospitals view environmental hygiene: not just as an area from which to cut costs, but one that can add value. Hospitals and key stakeholders must work together to change how we maintain the hospital environment in order to better protect patients.

## Introduction

Revolutions are often started by ideas whose time have come. Compared to other domains in medicine, revolutions in the field of Infection Prevention and Control (IPC) are generally few and far between. The last one was probably the global shift to using alcohol-based handrub (ABHR) instead of washing hands with soap and water, about 25 years ago- a seemingly small change in practice that continues to save millions of lives [1–3]. Today, looking at the IPC landscape, the one area that has been consistently undervalued and understudied is the role of the hospital environment in patient care. Keeping hospitals clean is not just an aesthetic, but a patient safety issue.

Although the available literature is limited, there is enough evidence to demonstrate that cleaning hospitals helps prevent infections. Still, good intervention studies are rare, the quality of products and methods available is heterogeneous, and environmental hygiene personnel is often relatively untrained, unmotivated, under-paid, and under-appreciated by other actors in the hospital. Coupled with understaffed environmental hygiene services departments, this creates lasting issues in regards to patient and healthcare worker safety.

The situation is not helped by the lack of literature concerning the exact impact that a soiled or contaminated environment has on healthcare-associated infections (HAI). In order to begin the initiative to change how hospitals think about their environment, a group of infection control experts teamed up with Interclean, the largest cleaning trade-show in the world, to create the Healthcare Cleaning Forum: a nexus where hospital managers, industry, and healthcare and cleaning experts could meet productively. For the first time, Interclean dedicated an entire hall to cleaning in healthcare, and hosted a conference featuring leaders in healthcare environmental science from across Europe. The speakers summarized the state of the art knowledge in the field, challenged the current dogma and began to shape a research agenda for developing the field of hospital hygiene and environmental control. This article outlines the major issues and points brought up during the conference. It attempts to illustrate the large gap that exists between environmental hygiene and the healthcare industry, as well as bring some much-deserved attention to a concept whose time has come.

### Cleaning as a patient safety initiative

**Fig. 1:**
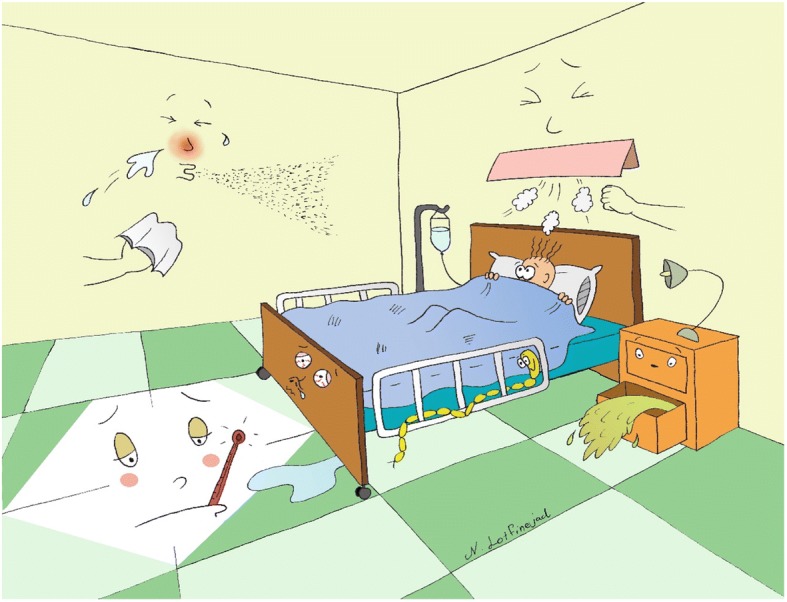
“No one wants to stay in a contaminated room”

We need to change how we think about the hospital environment- if the risks of transmission are known, no one wants to be the next patient in a contaminated room [4]. When the world changed how they thought about hand hygiene 25 years ago [2, 5, 6], it realized how important hands were as the main vectors for spreading diseases from one patient to another in hospital settings. It is estimated that over 50–70% of all HAI are spread through contaminated hands. It is time to focus on the other 30–50%, a part of which might be linked to environmental transmission (Fig. 1). After all, “hands are really just another highly mobile surface in healthcare that are commonly contaminated and rarely disinfected” [7]. Since there is a dynamic interchange between contamination on surfaces and hands [8], some of the transfer in which contaminated hands are the final link include contaminated surfaces as links earlier in the chain of transmission. Ideally, hospital environmental hygiene should follow the World Health Organization (WHO) model of “*Clean Care is Safer Care*” established for hand hygiene in 2005 [6], which spearheads good practices in more than 180 countries today [1, 9]. There is a need for creating evidence-based guidelines for hospital cleaning, and for using those guidelines to develop the right tools for education and implementation.

**Fig. 2:**
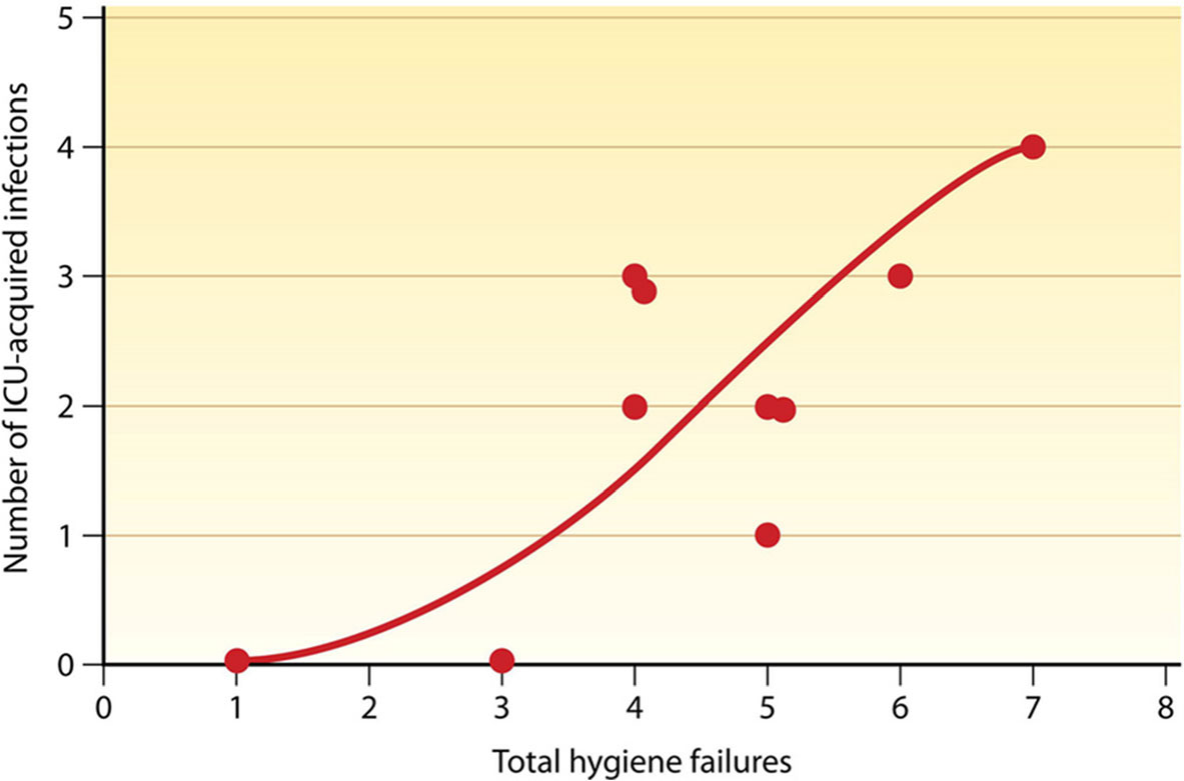
Relationship between environmental bioburden and hospital-acquired infection [10]

**Fig. 3:**
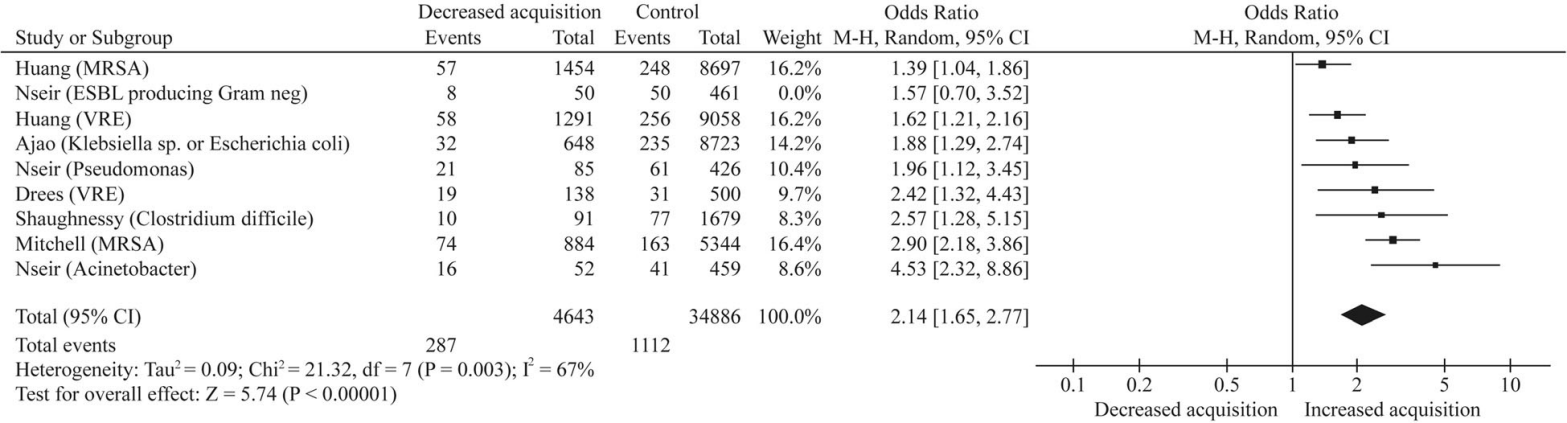
Risk of acquisition from prior room occupants by organism [4]. Risk of acquisition from prior room occupants by organism. M-H, Mantel–Haenszel; VRE, vancomycin-resistant enterococci; MRSA, meticillin-resistant *Staphylococcus aureus*; Ajao et al.’s study involved extended spectrum β-lactamase producing *Klebsiella* or *Escherichia coli* organisms. Acinetobacter: *Acinetobacter baumannii*; Pseudomonas: *Pseudomonas aeruginosa*. It was not possible to separate *Klebsiella* sp. and *Escherichia coli* data in the Ajao et al. study. Reprinted with permission from the Journal of Hospital Infection

Although high-quality interventional studies are limited, there are enough to show that cleaning and disinfecting hospitals in order to prevent infection works. Analysis of numerous studies shows a clear correlation between “cleaning hygiene failures” and the number of intensive care unit-acquired infections (Fig. 2). Several studies showed that patients were much more likely to contract certain pathogens if the patient in the room before them was colonized or infected with a pathogen linked to HAI (Fig. 3) [4, 10–12]. There is a wealth of information on what products or chemicals eliminate which pathogens and how to apply them. This includes efficacy and toxicity studies as well as a few clinical studies assessing the effect of specific interventions to control outbreaks [10]. More research is needed to measure the effects that cleaning methods have on HAI. Since improved environmental cleaning and decontamination measures are always bundled with other interventions during outbreaks, it is difficult to measure their precise impact.

### Cleaning in healthcare

Hospital environmental hygiene is complex because it is dependent on the pathogen present and the product used to remove it. There are five main variables to cleaning, whether removing soil or disinfecting and cleaning on a microbiological level (Table 1; the acronym “WASTE” can be used to recall the 5 variables). These elements are: what product or intervention is applied, the technique and equipment used to apply the product, the type of surface, the level of contamination of the environment, and last but not least, the environmental hygiene personnel doing the cleaning [13]. If any one of these elements is lacking, the cleaning will by definition be suboptimal. Because of this, changing cleaning practices in hospitals must be implemented through a multimodal strategy that takes these variables into account. The best cleaning substance in the world is useless if not applied correctly, and the best-trained personnel are useless if the product they are using is not effective against the particular pathogen that needs to be removed or killed.

**Table 1:** Environmental hygiene: How to get there – WASTE^a^

Workforce	The individuals responsible of organizing, executing and verifying a cleaning activity
Area	The environment to be cleaned. This includes the type of surface, if it is intact and the level and type of contamination.
Substance	The chemical component/product to cleaning, whether detergent or disinfectant
Technique	The method by which the cleaning substance is applied by either a person or a machine
Equipment	The machines or tools used to effectuate cleaning. This includes everything from a microfiber cloth to a hydrogen peroxide vapor machine.

^a^*WASTE:* workforce, area, substance, technique, equipment

But what is “clean”? (Table 2) Maintaining a hygienic hospital environment is not only about removing soil, but also about organizing an environment that is optimal for patient safety. Obviously if an area is visibly dirty, one cannot disinfect it. Visibly soiled surfaces must first be cleaned, and then, when or if appropriate, disinfected. Failure to do so means that the infective organisms cannot be targeted effectively [10]. The 2018 survey from the European network to promote infection prevention for patient safety (EUNETIPS) aimed to analyze how different hospitals evaluated and have created their cleaning strategies [14]. Cleanliness of a hospital also plays a large role in patient perception of the healthcare setting, and consequently of patient satisfaction [15, 16]. Patients are instrumental in convincing administrators to invest in cleaning, and must be sensitized to the issues in order to be allies for creating change.

**Table 2:** Glossary of terms

Term	Definition
Antisepsis	Destruction or inhibition of microorganisms in or on living tissue, e.g., on the skin or mucous membranes [29].
Automated disinfection	Disinfection using machines instead of manual application. Examples incl. Hydrogen peroxide vapor and UV light machines.
Cleaning	General term for the removal of soil.
Cleaning & disinfection	Removal of soil and killing of microbes.
Decontamination	The neutralization or removal of dangerous substances, radioactivity, or germs from an object, area or person [29].
Detergents	Water-soluble cleansing agents which combine with impurities and dirt to make them more soluble, and differ from soap in not forming a scum with the salts in hard water [30].
Disinfectants/disinfecting agents	Agents capable of destroying pathogenic microorganisms or inhibiting their growth activity [31]. *Especially*: chemicals that destroy vegetative forms of harmful microorganisms (such as bacteria and fungi) especially on inanimate objects but that may be less effective in destroying spores [32].
Disinfecting detergents	The combination of a detergent with a disinfecting agent for the simultaneous removal of soil and the killing of microbes.
Disinfection	The antimicrobial reduction of the number of viable micro-organisms to a level previously specified as appropriate for its intended further handling or use [29].
Environmental hygiene	Cleaning and/or disinfection of a specific environment.
Environmental hygiene personnel	People in charge of cleaning and disinfecting, and maintaining the hospital environment.
Environmental hygiene services	Service within a hospital that takes care of cleaning and hygiene of the environment.
Fumigation	To apply smoke, vapor, or gas especially for the purpose of disinfecting or of destroying pests [33]. In the past, this term was often used to mean automatic disinfection. In the context of environmental hygiene, the “pests” part of the definition does not usually apply.
Pasteurization	Disinfection, usually by heat, of microorganisms that can be harmful or cause product spoilage. Frequently applied for preservation. The prevention of the multiplication of microorganisms in products [29].
Resistance	The inability of an anti-infective or biocide to be effective against a target microorganism [29].
Sanitization	Disinfection of microorganisms that pose a threat to public health [29].
Sterilization	Defined process used to render a surface or product free from viable organisms, including bacterial spores [29]. It also frequently includes the objective of allowing the maintenance of the sterile state.
Terminal cleaning	Cleaning and disinfection of a room after a patient carrying a dangerous/resistant pathogen leaves the room.
Tolerance	Decreased effect of an active agent against a target microorganism and requiring increased concentration or other effects to be effective [29].

In General

a) Surfaces can be processed by detergent cleaning, disinfection, or a combination of the two (detergent plus disinfectant)

b) Medical devices require a pre-disinfection (immediately after use to prevent biofilm) including (or not) mechanical cleaning, followed by disinfection or sterilization

### Available products

In addition to a vast array of detergents and cleaning/disinfecting equipment, common chemicals used for disinfection include: alcohol, chlorine and chlorine compounds, formaldehyde, glutaraldehyde, hydrogen peroxide, iodophors, ortho-phthalaldehyde, peracetic acid, phenolics, and quaternary ammonium compounds [17]. This paper will not go into any detail on these products as such a discussion would be too extensive and was not the purpose of the Healthcare Cleaning Forum 2018.

An ideal product would be effective against all bacteria, spores, viruses, and prions while having no impact on the environment and being completely safe as well as easy to use. Currently no such product exists; thus choosing any product will result in some level of tradeoff.

For example, hydrogen peroxide vapor does not leave any residues in the environment, but is expensive, can be corrosive, and is difficult to use compared with liquid disinfectants. Chlorine solutions are effective against spores, but have a strong odor, leave residues, and may damage certain environmental surfaces. UV light leaves no residue but cannot disinfect areas that it cannot shine on directly. This is the case for every single product available today; most only work against certain types of pathogens, and others are toxic or degrade certain materials in the patient environment.

### The human component

But cleaning is not only dependent on the chemicals used. The ideal environmental hygiene personnel (Table 2) would remain thorough and meticulous, and always use the right technique, product and materials. Cleaning and disinfecting a hospital is a repetitive task that can quickly become mundane. Environmental hygiene personnel are often not trained sufficiently, and do not feel that they have the agency to make a difference in patient safety. Additionally, the amount of work that they are expected to do is not always in accordance with the time assigned to the task. Within the hospital hierarchy, environmental hygiene personnel are on one of the lowest rungs, and often credit is not given to them, especially considering the importance of their work. There is a major problem with how “cleaners” are often perceived as menial and uneducated by the rest of the hospital staff. In many countries, particularly in high-resources settings, cleaning personnel frequently originate from outside of the country, and do not express themselves in the local language, thus making discussions and interactive exchanges with other categories of health professionals difficult or even impossible.

Additionally, few hospitals have sufficient systems in place to train and certify their cleaning staff. Without certification, advancement is unstructured and can be limited since there is no way to gauge the quality of a staff as a whole. Often there is a high turnover rate among personnel within the cleaning service or a language barrier between the cleaners and the rest of the staff. Cleaning personnel must be trained to understand why their work is important to the hospital, and need to be recognized and certified in order to improve motivation and compliance [18].

### Logistics of hospital cleaning

The place of the environmental hygiene services department (Table 2) within a hospital is important, especially with regard to how they work together with the IPC service. Nursing assistants are generally responsible for cleaning one part of the patient environment and the environmental hygiene personnel for another; but often, respective tasks are not clearly defined. For example, if who needs to clean the bedside table is not explicitly stated, then there is a good chance that that table may not be cleaned by anyone. In one survey, one third of environmental hygiene personnel admitted that they were not really clear about what they were responsible for [19]. Absences or shortage of staff on wards, and/or the transfer of responsibilities between colleagues could complicate an already unclear situation and result in crucial maintenance not being performed. This can result in the spreading of disorder: a few minor mistakes, or disregard for a few of the rules, eventually cause increased disregard for rules in general among the whole staff [19].

In addition to the aforementioned issues, environmental hygiene services are often outsourced to external companies. While probably not as much of an issue in a stairwell or an office, it is virtually impossible for a hospital to optimize the cleaning staff and its quality if they have little to no oversight of or influence on the environmental hygiene. Outsourcing is not necessarily bad, but the right conditions must be observed, and crucial areas need to be cleaned by trained and certified professionals, even if costs are bit higher initially.

### Education, training and communication

So how can we effectively educate and train hospital personnel for modern environmental hygiene maintenance? While the science of cleaning and disinfecting agents and equipment has evolved immensely in the last few decades, the education of cleaning personnel and their integration into healthcare worker teams has not. Cleaning and disinfecting hospitals is very different from cleaning public spaces such as hotels or offices; hospitals must realize this and adapt to the challenges. There is a range of environments within each hospital, from offices to intensive care units or hospital pharmacy services, some of which require specialized approaches to environmental hygiene maintenance. There are even different requirements for different sectors within the same department. The pathogens present in hospitals can be quite different from those present in the community, and the patient population is more vulnerable. Each type of pathogen has its own specific transmission pattern, host affinity, and microbiological characteristics.

Leaders and trainers must be seen as legitimate by staff, and need to ensure their understanding and motivation. Only if there is a high process understanding in training can quality become routine; an informed person tends to be more compliant, and a compliant person is more motivated. Motivated teams are more efficient and more aware, and individuals need to understand that everyone’s work is important. Personal responsibility and team cohesion require solid collaboration, which in turn requires the equality and realization of rights and duties. Repetition, feedback and team-building help optimize performance in environments that inherently foster human error. Analyzing hospital architecture, workflow, and ergonomics can go a long way to reducing it. It is important to realize that the best product, equipment or intervention is worthless without well-trained, responsible and compliant staff.

### Possibilities of automation and self-disinfecting surfaces

Automation can be useful, but currently does not replace the need for thorough manual cleaning. Although manual cleaning and disinfection can be qualitatively as good as machine automated disinfection (or even better in some instances), one has less oversight over humans, and they do not clean at their best all of the time. Environmental hygiene service managers can use a variety of tools including visual inspection, cultures, ATP meters or UV light reactive fluorescent markers to verify how well a given area has been cleaned and disinfected. Though even the best-trained people are prone to error, machines never skip any steps. Automated or semi-automated room disinfection is not to replace personnel, but to raise the bar on the standard of disinfection and, in some instances, prevent work-related musculoskeletal constraints among environmental hygiene personnel. At some point, solely manual approaches are doomed to fail, as hospital environments are intricate and difficult to maintain in an appropriately clean state. In one study, up to 50% of an environment remained uncleaned after manual cleaning. Another study showed that after four rounds of manual cleaning and disinfection with a bleach solution, 25% of rooms were still contaminated with *Acinetobacter baumanii* [20]. Automated room disinfection with hydrogen peroxide vapor or ultraviolet light have shown promising results in targeting specific microorganisms, although they only work once a room has already been manually cleaned to remove soil [21].

Beyond machines, there is an important need for more research into surfaces that inherently inhibit bacterial contamination or that have self-disinfecting properties. A few that have been studied are the micro-patterning of surfaces or the inclusion of copper in them in order to reduce contamination [11]. The idea of having something permanently in the patient environment that is always working is an attractive one (although perhaps expensive): if one can control the level of contamination at the source, then there is less to remove and less risk for sub-optimal cleaning and disinfection. Further research, including unbiased, high-quality clinical efficacy and effectiveness studies are however still needed before further recommendations can be made regarding these materials [22, 23].

### Cost vs. value of hospital cleaning and disinfection

It is imperative to develop a new and efficient model for hospital environmental hygiene maintenance. The return on investment for successful hand hygiene promotion has been shown to up to 23 times the initial amount invested [24–26]. In order to have similar figures for hospital environmental hygiene, we need to first understand what the cost of maintaining a clean hospital environment is, and what its value is. Although many hospitals are quick to spend money on new software, specialized staff and fancy equipment, they often look at maintaining the environment hygiene as an opportunity to save in the budget.

**Fig. 4:**
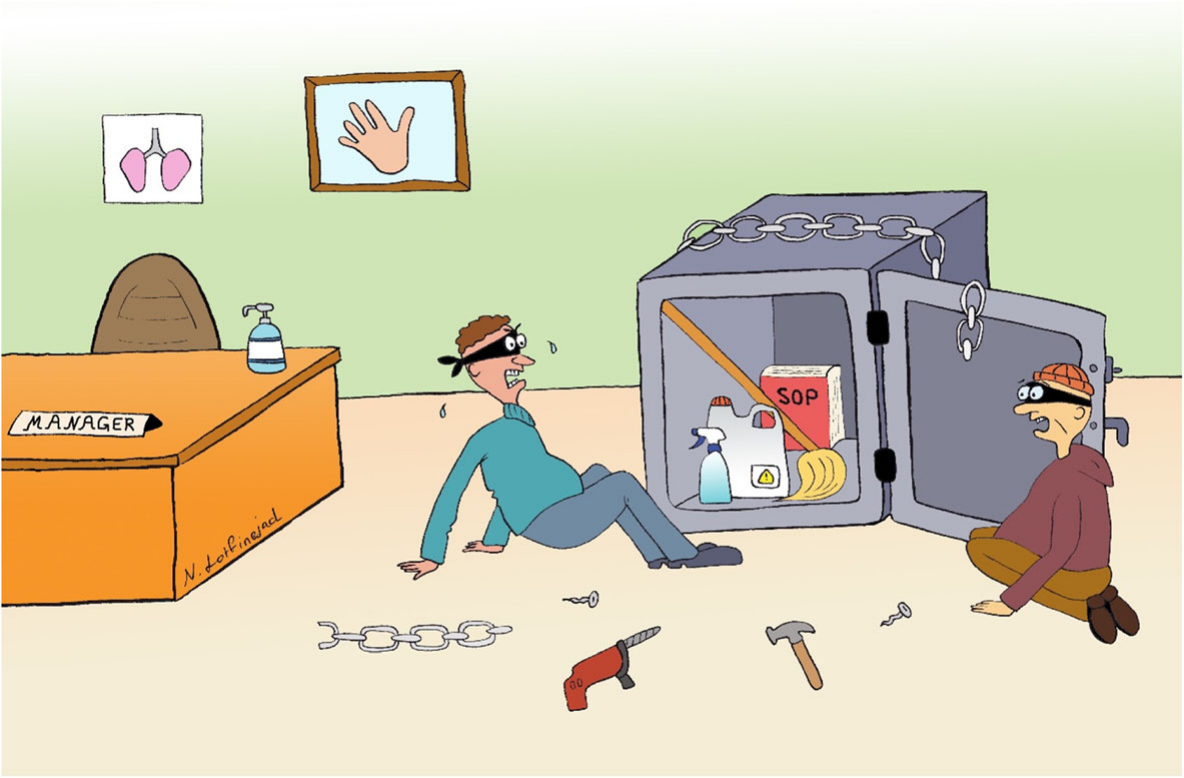
Hospitals should value environmental hygiene cleaning and maintenance

Hospitals often try to cut environmental hygiene maintenance costs as much as possible, both in the products that they use, and in the training and continued education of their workforce. The essential shift in approach needs to happen in how hospitals assess this cost and value. Because the costs of not cleaning can affect numerous budgets within a hospital, it is difficult to accurately account for them. Hospitals need to look beyond actual expenditures to averted expenditures, such as increase in patient-days due to HAI, as well as opportunity costs such as hospital staff time or missed surgical revenue due to increased turnaround time in an operating theater. There are also increases in costs associated with antimicrobial resistance in HAI, which has a cost estimated at over €85 trillion ($100 trillion) globally by 2050 [27]. For example, one relatively small outbreak with approximately 40 cases cost a hospital over €1 million [28]. Prevention is always better and less expensive than a cure, especially when we are running out of antibiotics. So when making a decision about which environmental hygiene maintenance systems to buy, which products to use, or how much to invest in training the cleaning personnel, hospitals would do well to look at the costs of not doing so, or deciding on a cheaper solution. In order to save money in the long-term and improve patient satisfaction, hospitals need to invest in quality across the board whether in materials, disinfectants, technological innovation, or the training, education, and certification of their workforce (Fig. 4).

### A time for cooperation

It is imperative to develop public-private partnerships in the field of clean hospitals. Industry and academia both have a role to play in raising standards and providing hospitals with the best possible products and methods. First, currently marketed sub-standards products and methods should be suppressed. Ultimately, the difference will no longer be between good and bad products on the market, but within implementation and training of those products and technologies.

Cleaning and environmental maintenance is a science. Initially, assessing the approach for the hygiene of a toilet seat seems almost redundant. However, many of the questions around this seemingly simple activity require study and scientific assessment. One must decide material to use to clean the toilet seat (e.g. microfiber or cotton cloth), which liquid agent to use (e.g. a detergent or a disinfectant and, if a disinfectant, which one), and the ideal frequency of the cleaning and disinfection (e.g. daily, after each use, or both). Many questions remain unanswered; some are addressed in the Appendix (see Appendix). Hospitals must get out of the vicious circle of cutting costs and instead assess value. They must realize that being a hospital “cleaner” is not a job but a profession, and invest in their workforce. Academics must encourage further studies (see research agenda, Table 3) as well as weave together the data available in order to present hospitals with a convincing business case of why to invest in hospital cleaning.

**Table 3:** Hospital cleaning: overall research agenda

Mobilize stakeholders	
Develop standardized guidelines for hospital cleaning	
Develop standardized operating procedures (SOP) for assessing quality of the cleaning performed	
Build a solid business case for investing in cleaning services, taking into consideration the cost and value of hospital cleaning and disinfection	
Encourage increased research in methods, implementation, compliance, and clinical outcomes of hospital cleaning	
Foster cooperation between private enterprise and public institutions	
Compile existing and publish new literature supporting the best products, technology and practices on the market	
Develop a model of a proven way of organizing hospital environmental cleaning services. Address the issue of cleaning personnel certification. How to train and educate personnel for modern cleaning tasks?	
Ensure process understanding in training: quality must become routine	
Address hospital perception of hospital environmental cleaning personnel- cleaning is not just a job, but a profession	

Because clean hospitals is an idea whose time is now.

### Comment

The authors alone are responsible for the views expressed in this article and they do not necessarily represent the views, decisions or policies of the institutions with which they are affiliated.

## Appendix

During the speaker presentations at the Healthcare Cleaning Forum conference in Amsterdam (May 16th, 2018), audience participants were able to ask questions directly via their smartphones. A total of 87 questions were submitted. Some of these questions were chosen and then posted on the screen above the speakers, who then addressed them directly after each presentation. These questions, as well as some that there were not posted during the conference, have been organized, and in some cases, combined or rephrased for clarity. The responses from the speakers have been synthesized in the table below.

Questions answered directly in the paper are not listed in the Table.

**Table 4:** Questions from the Healthcare Cleaning Forum 2018

	Questions on the technical process of cleaning:
Q:	In the process of wiping a surface clean, how many variables are involved?
A:	*Please see Table* *1* *.*
Q:	Is the use of probiotics allowed in the healthcare sector for cleaning and can this be a solution for difficult to reach surfaces?
A:	*Emerging evidence suggests that seeding the hospital environment with Bacillus spp. spores may reduce the level of pathogens that are culturable from surfaces. Whilst this could be as a result of competitive ecological exclusion, it could be possible that the Bacillus spores are merely masking the presence of pathogens. Further evaluations of this approach, including clinical outcome studies, are required.*
Q:	Is a combined disinfection and detergent wipe better?
A:	*It depends on the application. There are instances where it is better, but other instances where a disinfectant may not be required. Please see Table* *2* *.*
Q:	Are bacteriophage-based disinfection technologies (aerosolized for instance) considered as a complementary solution for disinfection?
A:	*Bacteriophage-based disinfection of the environment, whether applicable to surfaces or air, deserves both a literature search (particularly in Russian) and further in vivo and clinical research.*
Q:	Is it worth investing in an airborne disinfection solution?
A:	*There is emerging evidence that contaminated air may be involved in the transmission of pathogens that were traditionally associated with contact transmission, such as C. difficile and Acinetobacter spp. Further research is needed to understand the role of airborne transfer and airborne disinfection in hospital environmental hygiene.*
Q:	Does the cleaning of equipment wheels in ward areas help in reducing infection?
A:	*There is no evidence-based research demonstrating that cleaning of equipment wheels in regular ward areas helps to reduce infection; it is usually recommended at entrance of high-risk areas (ie. operative theatre), but further research is needed for definitive guidelines to be recommended.*
Q:	With no-touch cleaning and disinfecting, how is the soiling contamination removed? If the soil remains behind, is it possible to disinfect?
A:	*The room or area should be cleaned to remove dirt and organic soiling before an automated room disinfection system is applied. Please see also Table* *2* *for definitions.*
Q:	How do you deal with the issue of shadows in UV (ultraviolet light) systems?
A:	*Whilst the efficacy of UV systems in areas that are out of direct line of site of the UV device receive a lower dose of UV, they do receive a dose of UV due to reflection from other surfaces. The impact of line of sight in UV room disinfection can be mitigated by staging the device in different parts of the room, or using multiple emitters. The only solution is to change the angle of the UV light or to use alternative methods for decontamination.*
Q:	How important is cleaning of ceilings? How important is selection of building materials so that surfaces are less prone to infection?
A:	*Cleaning of ceilings is not that important as patients do not come in contact with ceilings. Surfaces must be chosen that are chemically resistant and easy to clean (non-porous). No surface is “resistant to infection”; some surfaces could be less prone to contamination. Please see also Table* *2* *for definitions.*
Q:	Have either hydrogen peroxide vapor (HPV) or UV-C devices been proven superior to the other in preventing surgical site infections?
A:	*To the best of the authors’ knowledge, none of the two methods has been associated with a significant reduction in surgical site infection in a controlled study.*
Q:	What are your thoughts about preventive (not corrective) disinfection with UV-C in high-risk areas after standard cleaning procedures?
A:	*Most studies that tested the impact of UV devices in healthcare settings used the devices to treat the rooms of patients known to be infected or colonized with a pathogen. There is a theoretical possibility that using UV more regularly would have an impact, but this requires further evaluation.*
Q:	What is your opinion on the overuse of chlorine and its health impacts in our hospital cleaning personnel?
A:	*On the one hand, we need chlorine, as it is one of the few active substances on spores, easily available and cheap. On the other hand, we can reduce the risk of respiratory and muco-cutaneous toxicity by always wearing appropriate protective equipment, using chlorine in the recommended concentration and only in the required situations. Viable alternative sporicidal agents to chlorine (such as peracetic acid and hydrogen peroxide-based chemistries) are now available, and should be considered.*
	Strategic Questions for Companies:
Q:	For an experienced cleaning vendor wanting to enter into the healthcare cleaning sector, what advice would you give to the company?
A:	*Get the best training in the field with infection control and hospital cleaning professionals.*
Q:	Is there a guide for presenting a business case for improving cleaning practices to hospital administrators?
A:	*There are a number of published papers that provide help and support with business case writing – the authors are happy to provide further information upon request.*
Q:	Making certified cleaning professionals will cost more money. How to convince finance people from hospital to prioritize quality of cleaning instead of the budget?
A:	*Professionals’ certification should be part of hiring conditions and over the long-term, not be associated with significant cost increases for the institution. Return on investment would be evident as soon as any adverse event linked to misuse of cleaning methods/techniques related to the absence of adequate training/certification would occur.*
Q:	Do you think the move towards biosurfactants and microorganisms in cleaning chemicals will affect the industry?
A:	*Most probably not. The use of products respectful of the environment will, however, gain momentum.*
	Strategic Questions for Hospitals/Institutions:
Q:	Can (and should) patients be educated so that they can assess the healthcare facility they are staying?
A:	*Patients’ participation in IPC is advocated and could help institutions to take actions (see paper).*
Q:	How can cleaning be validated without standard methods to measure cleanliness?
A:	*There are different methods to assess cleanliness, but no universal standards. Further research is needed to propose and promote universal standards.*
Q:	What is the best way to measure cleanliness in a hospital room, and is it in real-time?
A:	*Visual inspection, fluorescent markers, and ATP measurements can be used in real-time; bacterial cultures of an area take more time and use more resources.*
Q:	How do cleaning and disinfection affect the rates of urinary tract infections?
A:	*To the best of the authors’ knowledge, there is yet no study that relates a possible relation between surface cleaning and urinary tract infection rates.*
Q:	Soft surfaces like mattresses and stretchers are commonly damaged in healthcare; how important is surface integrity in infection prevention?
A:	*It is virtually impossible to clean a damaged surface. Surface integrity must be preserved if a surface is to be cleaned.*
Q:	What is the recommended practice to suppress *Clostridium difficile* spores from the hospital environment?
A:	*Most experts recommend the use of a sporicidal disinfectant such as chlorine, chlorine-containing, or peroxygen-based substances to clean rooms or wards hosting patients colonized or infected with C. difficile and to control C. difficile in hospitals.*
Q:	Do you have any good success stories or tips to help engage healthcare workers to work closely with cleaning service providers?
A:	*Yes, there are documented success stories, but there is no universal model yet.*
Q:	Should IPC teams train hospital cleaning personnel?
A:	*IPC team members should be involved in hospital cleaning personnel training, together with the key collaborators/head of the hospital environmental cleaning department.*
Q:	Would a fixed ratio of hospital cleaning personnel per hospital bed be a helpful key performance indicator?
A:	*Yes it could be a very useful (structure-level) performance indicator; however, one would need further research and optimal adjustment to develop and propose such a model.*
Q:	How could HPV and UV be implemented within mixed and open wards or in an ICU?
A:	*Although possible, their application could be quite challenging in conditions with high occupancy bed and rapid turnover rates because areas treated using HPV or UV need to be vacated by patients and staff.*
Q:	Is average patient length of stay aggregated on the basis of underlying morbidity a better measure of infection cost than solely monetary values?
A:	*Yes, indeed. Most estimates of the monetary impact of infections are centered on increased lengths of stay. There are however many additional aspects to include in cost-effectiveness analyses.*
Q:	What is the recommended time for cleaning single patient room, a 4 bed-room and a 6 bed-room?
A:	*There is no standard time. Models need to be developed and validated.*
Q:	What is your perspective of HPV and UV disinfection systems in improving bed turnaround time?
A:	*Both HPV and UV will extend bed turnaround time (HPV more so than UV). But, under defined circumstances, both HPV and UV have been associated with reduced heathcare-associated infections. Therefore, there may be a net improvement in patient throughput. More evidence should however be generated before recommendations could be established at large.*
Q:	How should the person responsible for the environmental services in a hospital be recognized/ should they earn more for becoming excellent at their job?
A:	*Training, permanent position, job recognition, certification, and job progress are essential to maintain motivation, as in other professions.*
Q:	Is there any data on the cost of bed disinfection per bed in any EU member country?
A:	*The cost of cleaning/disinfection will vary widely based on the methods used and the local approach to delivering cleaning and disinfection.*
	Broader Issues:
Q:	How do you educate people in the developing world about health care hygiene, where the level of literacy and awareness is so low?
A:	*The level of literacy is not the most important parameter in maintaining the hospital environment clean; hospitals in low and middle resources countries can be maintained at a very high level. As mentioned above training, a permanent position, job recognition, certification, and job progress are essential to maintain motivation, as in other professions.*
Q:	What is your key target for the next 12 months with regard to healthcare cleaning?
A:	*See the proposed research agenda (Table* *3* *). Not all points will be addressed over the next 12 months, but this is the direction in which we would like to develop the field.*
Q:	Would television ads be useful for increasing public awareness?
A:	*This approach certainly deserves to be tested.*
Q:	In the private sector, a person who is persistently non-compliant is disciplined. Why can’t this be done in healthcare settings?
A:	*It has been done, but is certainly rare. Evidence suggests that a “sticks and carrots” approach to improving human behavior works best, with incentivizing the good more effective than penalizing the bad.*
Q:	What are the top factors that lead to lower healthcare-associated infection rates in hospitals?
A:	*Successful hand hygiene promotion is the top priority, and has been associated with significant risk reduction. Prevention of device-associated and surgical site infections are certainly key priorities together with appropriate use of antimicrobials. Hospital cleaning is part of key strategies to reduce the bio-burden from the environment associated with the risk of cross-transmission and spread of multi-resistant organisms, linked to almost all infections in healthcare.*
Q:	Are we ready for new critical outbreaks like Ebola?
A:	*Pandemic preparedness has improved, informed by outbreaks such as the Ebola outbreak in West Africa, as recently demonstrated in the handling of the recent outbreak in RDC. Handling such risks however merits constant attention and adaptation of both patient care and environmental control protocols.*
Q:	Without any standardized and validated cleaning methods how can an infectious diseases specialist approve a cleaning contract?
A:	*There is definitively a need for universal, standardized and validated cleaning protocols, as discussed in the paper.*
Q:	Using ABHR instead of hand washing was a game changing strategy for hygiene. What is the game changer in surface cleaning in terms of chemical, process, materials, equipment, etc.?
A:	*Developing a model for the implementation and culture change of environmental cleaning best practices could constitute the solution.*
Q:	Is there a guide or reference on the scope of the work of healthcare workers and cleaning service providers?
A:	*To the best of the current authors’ knowledge there is no such universal guide; further development is needed.*
Q:	Should national healthcare system reimbursement schemes (such as the NHS) reward/promote prevention in hospital cleaning?
A:	*This tool might be part of a solution; yet one must first develop universal recommendations before one could propose such a tool.*
Q:	Do you think we can improve the human factor without investing more in training and monitoring hospital cleaning personnel?
A:	*No, training and monitoring is key to improving behavior.*
Q:	Can we automate the human factor improvements?
A:	*Understanding human factors is vital to improving human behavior. Automation can help in some situations; it cannot replace optimal behavior.*
Q:	What is your opinion on the report of the Dutch Health Council saying that there is a serious risk of bacteria getting resistant against disinfectants?
A:	*There is no evidence that microbes become resistant to most disinfectants at clinically meaningful levels. However, considering that resistance to antiseptics, as well as to antibiotics, antiviral-, antifunfals, and antiparasitic agents do exist, careful attention should be recommended for specialized research laboratory so that emergence could be traced as soon as possible if it would appear.*
Q:	When there is outbreak, it is often blamed on cleaning service providers not doing a good job. How can we change the perception of “teamwork” among all stakeholders?
A:	*Outbreak investigation and control is a challenge. Cross-transmission risk can be controlled most frequently by multimodal, multi-disciplinary interventions involving all health staff at multiple levels. Environmental control is key and most frequently cleaning services providers and/or personnel are accused of not doing an appropriate job. Although it is most frequently not the case, outbreaks associated with the lack of appropriate environmental control have been clearly identified.*
Q:	When will WHO guidelines be updated to adapt to new technologies?
A:	*There are currently no WHO guidelines on environmental control including recent and new technologies; the authors have no information regarding the possible update of WHO guidelines.*
Q:	Is it possible to get the presentations from today’s event?
A:	*Each of the presentations are available on the website of the Healthcare Cleaning Forum by clicking on the individual speakers* [33].
Hand Hygiene References *Hand Hygiene: Numerous questions on hand hygiene came up during the forum. Because this paper is not on hand hygiene in particular, there are a number of references below that contain all of the pertinent information.* [7, 34–38]	

## Abbreviations

ABHR: Alcohol-based handrub
HAI: Healthcare-associated infection
IPC: Infection prevention and control
WHO: World Health Organization
